# Optimization of Nanoencapsulation of *Codium tomentosum* Extract and Its Potential Application in Yogurt Fortification

**DOI:** 10.3390/md23040147

**Published:** 2025-03-28

**Authors:** Micaela Costa, Cristina Soares, Aurora Silva, Maria Fátima Barroso, Pedro Simões, Mariana Ferreira, Paula Gameiro, Clara Grosso, Cristina Delerue-Matos

**Affiliations:** 1 REQUIMTE/LAQV, Instituto Superior de Engenharia do Porto, Instituto Politécnico do Porto, Rua Dr. António Bernardino de Almeida 431, 4249-015 Porto, Portugal; 1200125@isep.ipp.pt (M.C.); mass@isep.ipp.pt (A.S.); mfb@isep.ipp.pt (M.F.B.); cmm@isep.ipp.pt (C.D.-M.); 2 Department of Analytical Chemistry and Food Science, Nutrition and Food Group (NuFoG), Instituto de Agroecoloxía e Alimentación (IAA)—CITEXVI, Universidade de Vigo, 36310 Vigo, Spain; 3 LAQV, REQUIMTE, Departamento de Química, Faculdade de Ciências e Tecnologia, Universidade NOVA de Lisboa, Quinta da Torre, 2829-516 Caparica, Portugal; pcs@fct.unl.pt; 4 LAQV, REQUIMTE, Departamento de Química e Bioquímica, Faculdade de Ciências da Universidade do Porto, Rua do Campo Alegre, 4169-007 Porto, Portugal; mariana.ferreira@fc.up.pt (M.F.); agsantos@fc.up.pt (P.G.)

**Keywords:** Box–Behnken design, subcritical water extraction, phytosomes, seaweeds, food fortification

## Abstract

Marine macroalgae are excellent sources of bioactive compounds recognized by their pharmaceutical and biomedical potential. A subcritical water extraction (SWE) was applied to the macroalga *Codium tomentosum*, and the extract was used to prepare phytosomes. A Box–Behnken design was applied to optimize the entrapment efficiency. These phytosomes were further modified with DSPE-PEG (2000)-maleimide and apolipoprotein E and characterized by dynamic light scattering, UV spectrophotometry, octanol/water partition coefficient, differential scanning calorimetry, and Fourier transform infrared spectroscopy. As proof of concept, prototypes of functional food tailored to the elderly were produced. Yogurts were fortified with seaweed extract or phytosomes, and physicochemical properties and proximal composition (pH, acidity, syneresis, moisture, peroxides, proteins, total lipids, sugar content, ash, and mineral composition) were analyzed. The antioxidant and the inhibition capacity of two brain enzymes, cholinesterases (AChE and BuChE), involved in the pathogenesis of Alzheimer’s disease, were also evaluated in the final prototypes. Despite their unappealing sensory characteristics, the results are promising for integrating marine extracts with potential neuroprotective effects into functional foods.

## 1. Introduction

Due to the increase in life expectancy, promoting active aging is crucial in the 21st century [1]. Therefore, searching for new neuroprotective drugs is urgent. Alzheimer’s disease (AD) is the most prevalent neurodegenerative disorder, followed by Parkinson’s disease (PD) [2]. Most people diagnosed are 60 years old or older. For people aged between 65 and 69, around 2 in every 100 people have dementia, with AD being the most common form. A person’s risk then increases as they age, roughly doubling every five years [3]. Regarding PD, only 5 to 10 percent of people with this disease are diagnosed before the age of 50 [4].

Biris-Dorhoi [5] has reported a considerable variety of bioactive compounds identified in seaweed that may help prevent and treat many diseases. Seaweed consumption has been associated with a lower incidence of different chronic diseases, such as cancer, diabetes, neurodegenerative diseases, obesity-related disorders, and metabolic syndrome. Regarding specifically the neuroprotective activity of seaweeds, a broad class of compounds, such as polysaccharides, proteins, lipids, pigments, and polyphenols, isolated from macroalgae, have shown neuroprotective effects through mechanisms such as enzyme modulation or protection against amyloid-β (Aβ) or Tau-induced toxicity [6,7]. In AD patients, the degeneration of central cholinergic neurons reduces choline acetyltransferase (ChAT) activity, decreasing acetylcholine (Ach) synthesis. At the same time, increased cholinesterase activity accelerates Ach breakdown, worsening this neurotransmitter deficiency. In AD, AChE activity drops by up to 45%, but BuChE activity can double, making this cholinesterase a more significant target in the later stages of the disease. Thus, enhancing ChAT activity and reducing AChE and BuChE functions has become a critical strategy in AD treatment. Likewise, disrupting the balance between α-secretase and non-α-secretase pathways leads to increased BACE1 activity, resulting in Aβ oligomers and plaque accumulation, making BACE1 activity a critical therapeutic target in the AD treatment. Although tau protein hyperphosphorylation is an important therapeutic target for AD, only a few compounds have been identified that can effectively target tau protein [8].

The most promising result from seaweeds to date was achieved with sodium oligomannate, a mixture of oligosaccharides isolated from the marine algae *Ecklonia kurome* Okamura. Clinical trials have shown that it is safe and effective in treating mild-to-moderate AD, leading to its approval by the National Medical Products Administration (NMPA) of China in 2019 [9,10]. Recent studies suggest its neuroprotective effects are mediated through the microbiota–microglia–amyloid axis. It modulates gut microbiota while directly inhibiting Aβ fibril formation and neuroinflammation. Despite its therapeutic potential, the absorption, distribution, metabolism, and excretion (ADME) profile of sodium oligomannate remains poorly understood due to its complex high-molecular-weight and highly polar carbohydrate structure. Further research is necessary to elucidate its systemic bioavailability, brain penetration, and precise mechanisms of action in neurodegenerative diseases [9,11].

In addition, Peñalver et al. [12] described several studies reporting that incorporating seaweed and/or seaweed extracts into food products improves properties such as the shelf life, nutritional content, and textural and sensory aspects of the final products. However, some of these effects differ according to the seaweed species and the amount used in the formulation.

*Codium tomentosum* is a green macroalga with neuroprotective potential, as reported by Silva et al. [13], who have shown that several fractions obtained from the methanolic and dichloromethane extracts of *C. tomentosum* displayed neuroprotective activity in thrice-subcloned SH-SY5Y cells line exposed to the neurotoxin 6-hydroxydopamine (6-OHDA). The authors also investigated which mechanisms were triggered by these fractions, concluding that they mitigated reactive oxygen species (ROS) generation, counteracted mitochondrial dysfunctions and DNA damage, and reduced caspase-3 activity. Recently, a fraction obtained by subcritical water extraction (SWE) in the range of 190–250 °C was tested against several brain enzymes involved in the etiology of neurodegenerative and neuropsychiatric disorders, namely, cholinesterases, monoamine oxidase A and B and tyrosinase, revealing moderate inhibition (IC_50_ [AChE] = 239.8 µg/mL, IC_50_ [BuChE] = 430.1 µg/mL, IC_50_ [MAO-A] = 181.2 µg/mL, IC_50_ [MAO-B] = 422.5 µg/mL, and IC_50_ [tyrosinase] = 15.3 µg/mL). The same fraction was also demonstrated to be a strong superoxide radical anion (O_2_^•−^) and nitric oxide radical (^•^NO) scavenger (IC_50_ = 85.7 µg/mL and 254.2 µg/mL, respectively) [14].

To counteract the low absorption of bioactive phytocompounds, nanosized drug delivery systems like phytosomes can enhance their penetration across biological barriers, such as the blood–brain barrier (BBB), thereby promoting their delivery to the desired target [15]. Phytosomes are innovative lipid-based nanocarriers with liposome-related structures but with some additional advantages. While hydrophilic biomolecules are just entrapped in the aqueous core of liposomes without chemical bonds being formed, in phytosomes, H-bonds are established between the extracts and the phosphate group of phospholipids, increasing their stability [15]. This approach has been successfully applied to encapsulate several plant-derived bioactive compounds [16].

While using natural extracts in functional food development is growing, incorporating seaweed or seaweed extracts remains problematic—mainly due to their strong sensory characteristics and limited consumer knowledge of their potential health benefits. Furthermore, regulatory requirements for substantiating health claims often necessitate preclinical or clinical evidence, adding complexity to commercialization efforts [17]. Luckily, new milk products have been produced by adding seaweed extracts and the impact on the product’s final appearance has been evaluated. Roohinejad et al. [18] reported that the greenness and yellowing of fortified milk samples increased by adding ethanolic extracts prepared from seaweed also provided antioxidant functionality. Moreover, the type or concentration of seaweed extract did not influence quality parameters, e.g., microbiology. The sensory evaluation showed that the extract was accepted as a functional ingredient in milk. Seaweed extracts were found to be stable in milk, showing that their addition can improve certain milk qualities and shelf life characteristics.

This work aimed to extend the study developed by Soares et al. on the assessment of the bioactivities displayed by different subcritical water extraction (SWE) fractions of *C. tomentosum* [14], by designing and characterizing phytosomes containing the most active fraction, herein called *C. tomentosum* extract (CtE), to incorporate it into a neuroprotective functional food product—yogurt—targeted at senior citizens, who are at increased risk for neurodegenerative and neuropsychiatric disorders. The CtE (obtained through SWE at 190–250 °C, 100 bar) is abundant in phenolic compounds and Maillard reaction products (MRPs) [14]. Both classes of compounds are known for their antioxidant and neuroprotective properties [19,20,21], However, due to the high diversity of MRPs, some MRPs, such as acrylamide, toxic advanced glycation end products (TAGEs), and heterocyclic amines, have been implicated in the development of several diseases [22,23,24].

The phytosomes that were generated underwent characterization to identify key characteristics like size and composition. Furthermore, the nutritional profile of the food prototype, along with its antioxidant (DPPH^•^ and ABTS^•+^ scavenging activities and oxygen radical absorbance capacity (ORAC) assay) and neuroprotective (AChE and BuChE inhibition) properties, was assessed as well.

## 2. Results and Discussion

### 2.1. Box–Behnken Design

The entrapment efficiency of CtE varied according to the set of conditions tested, ranging from 25.19% (*A*—1 h, *B*—42.5 °C and *C*—1:4) and 45.70% (*A*—2.5 h, *B*—25 °C and *C*—1:4; and *A*—1 h, *B*—60 °C and *C*—1:2.5) (Table S1).

**Figure 1:**
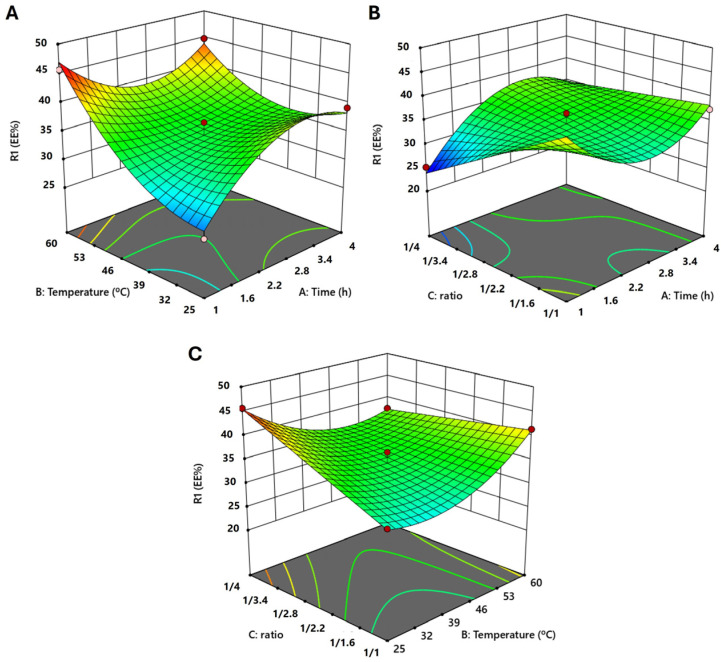
Three-dimensional surface graphics. (**A**)—Temperature vs. Time; (**B**)—Ratio vs. Time; (**C**)—Ratio vs. Temperature.

A reduced cubic model was fitted to the experimental results, and the most significant parameters (*p* < 0.05) were *A*, *C*, *AB*, *AC*, *BC*, *B*^2^, *A*^2^*B*, and *A*^2^*C* (Table S2, Figure 1). The resulting model equation (Equation (1)) was:

(1) $$\begin{array}{c} \mathrm{Entrapment}\;\mathrm{efficiency}\left(\%\right)\\ \;\;\;\;\;\;\;=29.12782+4.76425A+0.428789B-9.71050C-0.873397AB+13.17704AC-0.167143BC\\ \;\;\;\;\;\;\;-0.406866A^{2}+0.014842B^{2}+0.148889A^{2}B-2.31630A^{2}C \end{array}$$

With the reduced cubic model, the calculated coefficients of determination (*R*^2^), adjusted *R*^2^, predicted *R*^2^, and adeq precision were, respectively, 0.9666, 0.8832, 0.4041, and 12.1256. The model was significant (*p* = 0.0153), and the lack of fit was not significant (*p* = 0.4916) (Table S1). The diagnostic graphics (Figure S1) show a good fit of the model to the experimental results.

Two optimal conditions (Derringer’s desirability function = 1.000) were retrieved from the model to obtain the highest % of complexation: condition 1 (*A*—1 h, *B*—59 °C, and *C*—1:1) and condition 2 (*A*—4 h, *B*—25 °C, and *C*—1:4), achieving a predicted value of 58.92% and 54.65%, respectively. At these optimal conditions, experimental values obtained were 61.76 ± 10.11% (*n* = 7) and 57.63 ± 10.65% (*n* = 7), respectively, showing that the model predictions were quite close to the actual values.

The optimal conditions for preparing phytosomes, as determined from the model, were consistent with those reported in previous studies [25,26,27]. Telange et al. [25] tested different conditions to prepare phytossomal complexes with apigenin, namely, different molar ratios of apigenin and phospholipid (1:1, 1:2, and 1:3) and temperatures (40, 50, and 60 °C). The reaction occurred during 2 h in a mixture of 1, 4–dioxane:methanol (14:6). A full-factorial design (3^2^) was applied, revealing that a ratio = 1:2, and T = 60 °C were the optimal conditions to achieve the highest complex formation (93.7%). In another study, ginger or ginger plus rosehip and phosphatidylcholine (PC) were weighed in different ratios, dissolved in ethanol, and refluxed at 60 °C for 2 h. To determine the encapsulation efficiency of the phytosomal formulation, the non-entrapped free 6-gingerol and β-carotene were separated from the phytosomal complex by ultracentrifugation. The encapsulation efficiency of 6-gingerol and β-carotene in all preparations was between 83.3% and 94.3%. Although phytosomes containing 0.9 of ginger extract:0.1 of rosehip extract: 1 of PC displayed lower particle sizes (193.72 nm), phytosomes prepared with a ratio of 0.5:0.5:1 were chosen for further assays due to their strongest anti-inflammatory and antioxidant effects [26]. On the other hand, using room temperature was favorable for producing phytosomes with *Diospyros kaki* L. acetonic extract (acetone:water (80:20, *v*/*v*)) [27]. The authors mixed the extract with PC in a molar ratio of 1:1 or 1:2, in ethanol, for 2 h at 25 °C, obtaining entrapment efficiencies above 90%.

### 2.2. Characterization of Phytosomes

The phytosomes were functionalized before characterization. The DSPE-PEG (2000) peptide was employed to enhance the targeting specificity of the formulated nanoparticles, helping them evade digestive processes and immune responses [28], while ApoE was used to guide the nanoparticles to the BBB [15].

Concerning their size and polydispersity index (PDI), phytosomes produced through condition 1 (*A*—1 h, *B*—59 °C, and *C*—1:1) showed to be lower and less dispersed than those produced through condition 2 (*A*—4 h, *B*—25 °C, and *C*—1:4)—245.76 ± 49.00 nm and PDI = 0.26 ± 0.06 vs. 261.02 ± 70.46 nm and PDI = 0.32 ± 0.02, respectively. Therefore, condition 1 was selected for further characterization. This behavior is in line with the results reported by Direito et al. [27] who observed that phytosomes prepared with a ratio of 1:2 (extract:PC) tended to agglomerate more than those prepared using a ratio of 1:1 (193.3 nm and PDI = 0.347 vs. 177.1 nm and PDI = 0.184, respectively).

The study of the solubility in water and in n-octanol of the CtE and of the phytosomes produced showed that the isolated extract had high aqueous solubility (95%) and low solubility in n-octanol (5%), which would be expected due to the highly aqueous character of the extract. On the other hand, the complexation of the extract with PC conferred an amorphous character to the system and moderated its lipophilic character. This resulted in improved solubility in n-octanol and dissolution profile. This way, an increase in lipophilicity to 28% was observed for the phytosomes, resulting in a Kow = 0.382. These results align with previous studies reporting that phyto-phospholipid complexes have better lipophilicity and hydrophilicity than their active constituents [29,30]. Typically, these complexes demonstrate enhanced lipophilicity, which offers a significant advantage for the food and pharmaceutical industries in delivering polar compounds [29,30].

**Figure 2:**
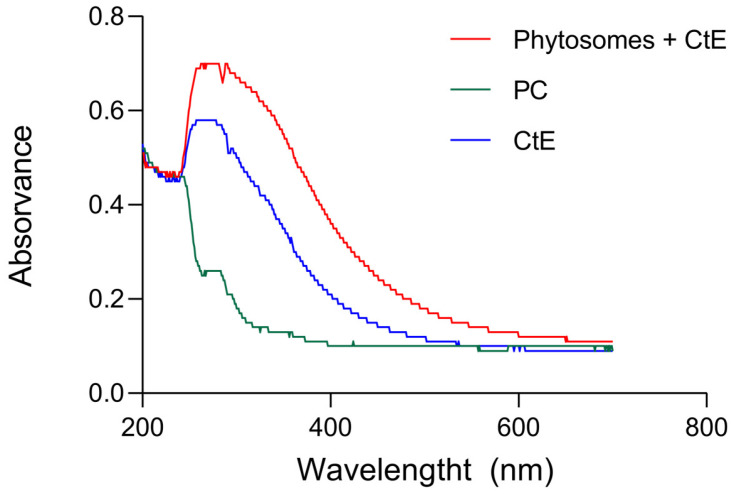
Spectra of samples in the UV and the visible spectrum [Blue] CtE; [Green] PC; [Red] Phytosomes containing CtE.

The UV spectra of the CtE and phytosomes were analyzed in the 200–700 nm range. As shown in Figure 2, the characteristic absorption peak of the CtE (280 nm) remained present in the phytosome, suggesting that weak physical interactions between the extract and PC occurred during the formation of the complex.

**Figure 3:**
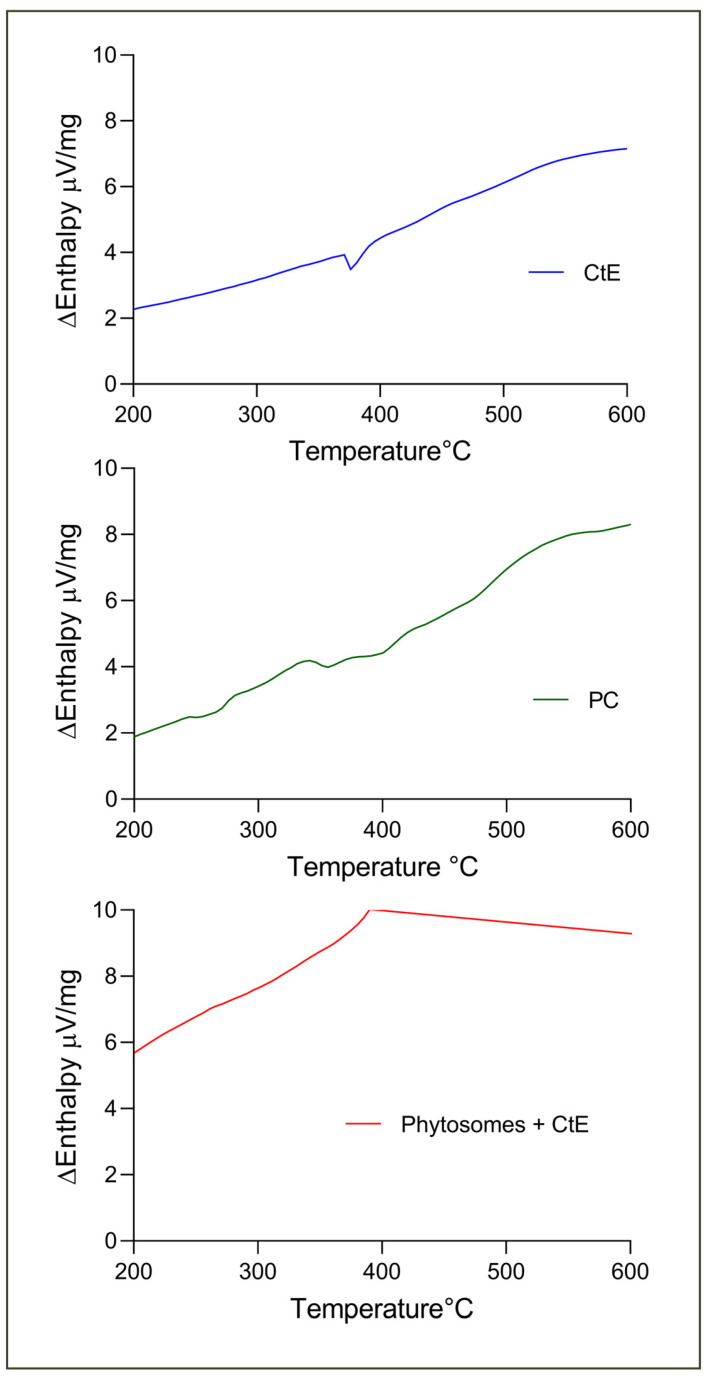
Differential scanning calorimetry spectra of samples: [Blue] CtE; [Green] PC; [Red] phytosomes with CtE.

The thermograms of CtE, PC, and phytosome are represented in Figure 3. The extract exhibited a sharp endothermic peak at 377.5 °C corresponding to the sample melting. PC presented three low-intensity peaks (249.5, 355.8, and 391.4 °C) related to the fusion of the polar region and the melting of the hydrophobic tails of the phospholipids [30,31]. The phytosomal complex presents a new peak, completely different from those observed in the individual constituents of very low intensity at 462.5 °C, which could be related to the appearance of new hydrogen bonds between the -OH groups of the phenolic rings of the CtE with the phospholipid [30,31].

**Figure 4:**
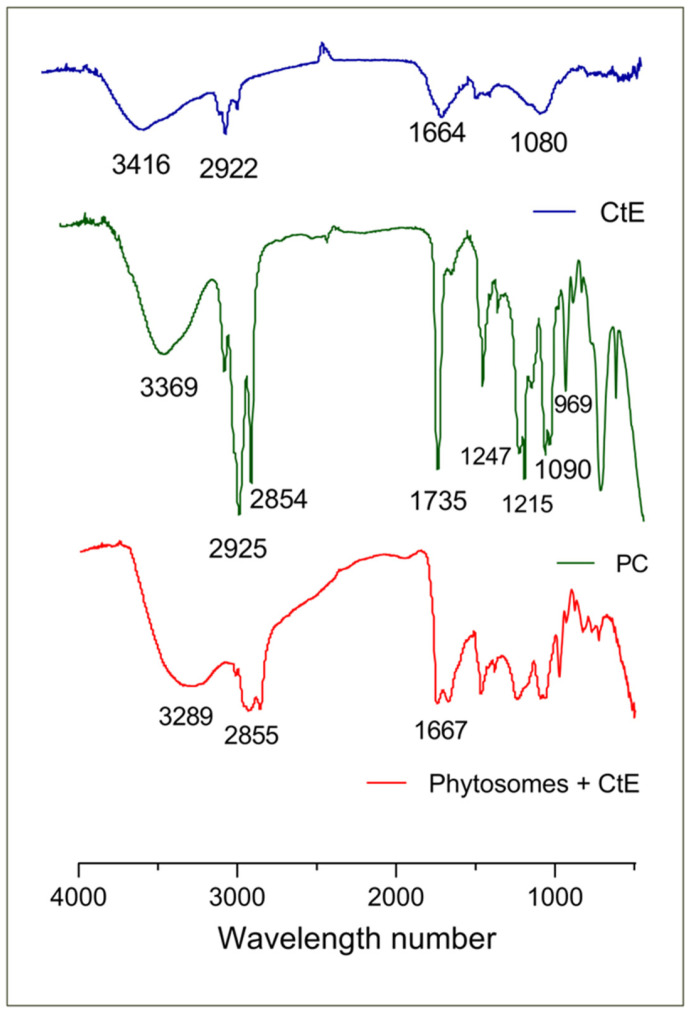
Fourier transform infrared spectroscopy of samples: [Blue] CtE; [Green] PC; [Red] phytosomes with CtE.

The possible interaction between the CtE and PC in the phytosome was studied by FTIR (Figure 4). The peaks at 3416.62, 3369.38, and 3289.81 cm^−1^ represent hydroxyl (OH) stretching in the IR region of CtE, L-α-phosphatidylcholine, and the phytosome complex, respectively (Figure 4). The absorption signals at 2922.01 cm^−1^ of the CtE and 2854.92 cm^−1^ of the complex represent the characteristic C-H stretching of the long chain of fatty acids, and the peaks at 1664.39 cm^−1^ of the CtE and 1667.26 cm^−1^ of the complex represent the C=O stretching, also alluding to the existence of aromaticity [32]. In the case of the CtE, a peak appears at 1079.99 cm^−1^ for the ether group (C-O-C) [32].

The FTIR spectrum of PC (Figure 4) marked the presence of multiple characteristic absorption signals: at 2925.81 and 2854.71 cm^−1^, representing the characteristic C-H elongation band of the long fatty acid chain; at 1735.17 cm^−1^, a characteristic of the carbonyl stretching of the fatty acid ester; at 1247.26 and 1215.94 cm^−1^, corresponding to P=O stretching band (a characteristic of the phosphate functional group); at 1090.92 cm^−1^, representing P-O-C elongation; and at 969.12 cm^−1^, due to N-+(CH_3_)_3_ stretching [31,32,33].

No new significant peaks were observed in the complex (Figure 4). However, the C=O peak of the CtE was shifted in the complex to higher wavelengths, and there was also a clear absorption band at the 1234.51 cm^−1^ characteristic, in the case of complexes, of strong C–O bonds, proving the formation of new bonds. There was also a broadening of the characteristic phenolic band (OH) caused by the formation of hydrogen bonds, indicating that the phytosome formulation was successful [30].

### 2.3. Characterization of Fortified Yogurt

An initial sensory screening was conducted to eliminate yogurt formulations with undesirable organoleptic attributes. This step allowed us to focus on subsequent analyses (physicochemical, antioxidant, and bioavailability) of the most acceptable formulations in terms of taste, texture, and appearance.

#### 2.3.1. Sensorial Analysis and Selection of Optimal Formulations for Further Analysis

**Figure 5:**
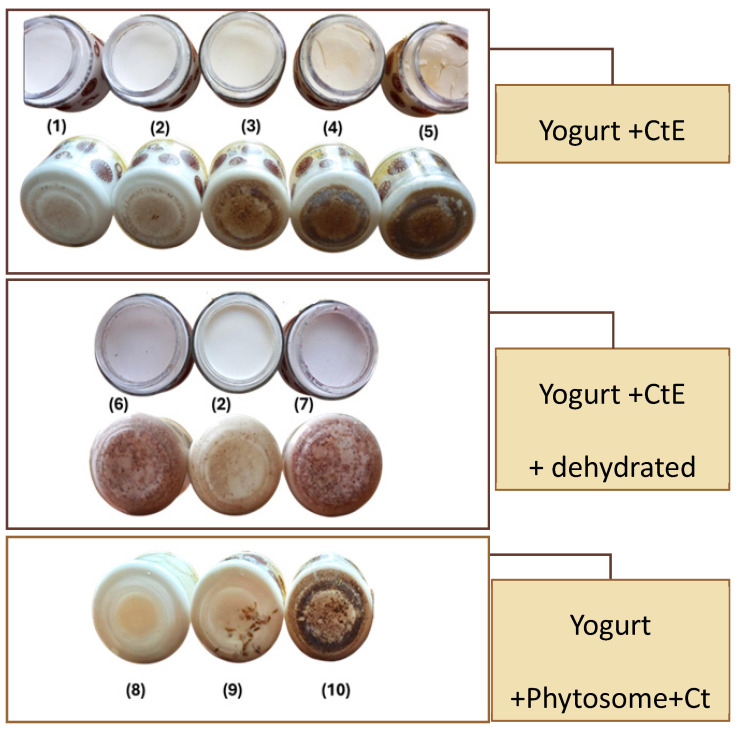
Visual appearance and sedimentation patterns of yogurts formulated with varying concentrations of CtE and phytosome-encapsulated CtE. Top section: Yogurts with 0.005% (1), 0.01% (2), 0.05% (3), 0.10% (4), and 0.2% (5) CtE, showing increasing surface cracking and instability at higher concentrations. Middle section: Yogurts with 0.01% CtE with (6) and without (2) dehydrated fruit and 0.15% CtE with dehydrated fruit (7), highlighting the masking effect of fruit on sedimentation. Bottom section: Yogurts with 0.01% (8) and 0.15% (9) CtE in phytosomes, as well as 0.15% unencapsulated CtE (10), demonstrating the effectiveness of phytosome encapsulation in reducing sedimentation at lower concentrations.

Visual evaluation of yogurt samples (Figure 5) revealed that both the concentration and form of CtE significantly influenced surface texture, color, and sedimentation patterns. At low CtE concentrations (0.005–0.01%), yogurts maintained a smooth, uniform surface and minimal sedimentation, comparable to the control. However, at ≥0.05% CtE, surface cracking, gel destabilization, and visible sedimentation became evident, particularly at 0.2%, likely due to the interference of the extract’s high polysaccharide and mineral content with the yogurt matrix [34]. Additionally, sedimentation was increasingly visible at higher concentrations, with dark deposits accumulating at the bottom of the containers. Yogurts containing more than 0.05% CtE fraction exhibited a yellowish, unappealing hue, similar to findings from previous studies on seaweed extract incorporation in yogurt [35].

To counter these effects, two strategies were tested: phytosome encapsulation and dehydrated fruit addition. Phytosome encapsulation improved CtE dispersion and phase stability, particularly at 0.01%, where yogurts exhibited a homogeneous structure with minimal sedimentation. Even at 0.15% CtE, encapsulated samples showed reduced aggregation compared to their unencapsulated counterparts, confirming the technique’s effectiveness [35,36,37]. Nevertheless, formulations with 0.15% CtE showed structural instability, indicating the potential need for hydrocolloids or stabilizers in future developments.

Dehydrated blackberry or raspberry enhanced visual appeal by masking sedimentation, likely due to particle coverage, which may also increase consumer acceptability [38]. Sensory analysis showed that CtE concentrations ≥ 0.10% increased acidity, partially balanced by fruit addition, while aroma remained consistent with standard yogurt.

Other studies using higher seaweed concentrations reported strong seaweed flavor and low flavor quality (e.g., 0.5% *Undaria pinnatifida* or *Saccharina latissima*) [39]. In contrast, our formulations using ≤0.15% CtE demonstrated moderate sensory impact and improved integration when paired with phytosomes or fruit. However, 0.15% CtE, samples still showed structural instability, suggesting the potential need for hydrocolloids or stabilizers.

Based on appearance, flavor, and texture, the following formulations were selected for further analysis: control yogurt (0% CtE); 0.01% CtE (unencapsulated); 0.01% CtE in phytosomes; 0.01% CtE + dehydrated fruit; 0.15% CtE; and 0.15% CtE + dehydrated fruit.

#### 2.3.2. Inhibition Capacity of Brain Enzymes Cholinesterases (AChE and BuChE)

The control sample (yogurt without CtE) showed no or weak inhibition of cholinesterases. This result is in accordance with the study performed by Ramadan et al. [40]. These authors investigated the influence of three different treatments to reverse the neurotoxicity induced by AlCl_3_ in rats: plain yogurt, microcapsules of triple omega 3-6-9, and fortified yogurt with those microcapsules. They concluded that treatments with fortified yogurt and microcapsules were more effective than plain yogurt in reverting the increase in AChE and BuChE activity promoted by AlCl_3_.

**Figure 6:**
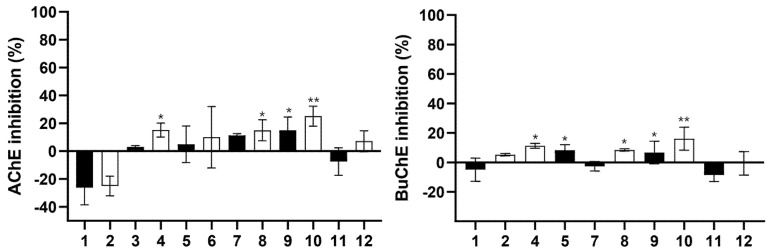
AChE and BuChE inhibition. 1—Control yogurt (2 mg/mL); 2—Control yogurt (4 mg/mL); 3—Yogurt with phytosomes 0.01% (2 mg/mL); 4—Yogurt with phytosomes 0.01% (4 mg/mL); 5—Yogurt with CtE 0.01% (2 mg/mL); 6—Yogurt with CtE 0.01% (4 mg/mL); 7—Yogurt with CtE 0.01%+ dehydrated fruit (2 mg/mL); 8—Yogurt with CtE 0.01%+ dehydrated fruit (4 mg/mL); 9—Yogurt with CtE 0.15% (2 mg/mL); 10—Yogurt with CtE 0.15% (4 mg/mL); 11—Yogurt with CtE 0.15%+ dehydrated fruit (2 mg/mL); 12—Yogurt with CtE 0.15%+ dehydrated fruit (4 mg/mL). Bars represent mean values, with significance indicated by * and ** for *p* < 0.05, representing samples that differ significantly from others.

Low AChE and BuChE inhibition percentages were obtained due to the low concentration of Cte or phytosomes incorporated in the yogurts (Figure 6). The best results (30% AChE inhibition) were shown in the yogurt containing 0.15% of CtE (4 mg/mL of sample) (Figure 6).

The percentages of inhibition obtained are relatively low due to the yogurt manufacturing integrating rather low percentages of the CtE and phytosomes. However, we have reported that the CtE shows a high percentage of inhibition of cholinesterases [14]. Therefore, by the cumulative effect, our results support that the possibility of achieving improved inhibition should not be discarded.

#### 2.3.3. Antioxidant Activity

The prepared yogurts’ radical scavenging ability was measured using the ABTS^•+^ and ORAC assays. The results are presented in Table 1.

Table 1 shows that the control yogurt had the lowest antioxidant activity (ABTS: 13.5 mg TE/100 g; ORAC: 62.1 μmol TE/100 g), providing a baseline. Yogurt containing 0.01% CtE in the phytosome form showed no significant improvement, suggesting a limited release of bioactives due to encapsulation. This may reflect strong interactions with yogurt components, stabilizing the formulation but limiting an immediate antioxidant action.

In contrast, adding 0.01% unencapsulated CtE markedly increased ORAC values (118 μmol TE/100 g), while ABTS remained like the control—implying assay sensitivity differences. When dehydrated fruit (DF) was added to this formulation, both ABTS (24.2 mg TE/100 g) and ORAC (245 μmol TE/100 g) increased significantly, suggesting a synergistic effect between fruit and seaweed-derived polyphenols. A higher CtE concentration (0.15%) further increased antioxidant values, especially ABTS (27.0 mg TE/100 g), though ORAC peaked at 0.01% CtE + DF.

Previous research has demonstrated the potential of seaweed extracts, such as those from *Ascophyllum nodosum* and *Fucus vesiculosus*, to enhance yogurt’s antioxidant properties and shelf life. In a study where yogurts were formulated with 0.25% and 0.5% seaweed extracts, improvements in radical scavenging activity measured using the DPPH assay, lipid oxidation stability, and microbiological quality were observed over a 28-day storage period. The yogurt containing 0.5% *F. vesiculosus* extract exhibited a significantly higher radical scavenging activity (*p* < 0.05) compared to the control, reinforcing the role of seaweed bioactives in improving yogurt functionality [35].

#### 2.3.4. pH, Titratable Acidity, and Peroxide Value

The pH, titratable acidity, and peroxide values of yogurt samples were evaluated during 7 days of storage at 4 °C to assess the impact of the CtE, phytosome encapsulation, and dehydrated fruit addition on yogurt stability over time. The results are presented in Table 2.

Initial pH (pH_0_) values ranged from 4.26 to 4.40 (*p* < 0.05). The highest (4.40 ± 0.01) was observed in phytosome-encapsulated 0.01% CtE, suggesting delayed acidification. The lowest pH (4.26 ± 0.01) occurred in 0.01% CtE + DF, likely due to fruit-derived organic acids. These values are comparable to those in seaweed-fortified yogurts from previous studies [41].

By day 7 (pH_7_), all samples showed a slight pH decline, with values ranging from 4.23 to 4.37 (*p* < 0.05). The control yogurt dropped from 4.36 to 4.31, showing a 1.2% reduction, while CtE- and phytosome-fortified samples exhibited smaller pH decreases (~0.7%), suggesting that phenolic compounds may inhibit bacterial metabolism and slow acidification [42,43]. The highest final pH (4.37 ± 0.01) remained in the phytosome sample, reinforcing its stabilizing effect on microbial activity. The lowest (4.23 ± 0.01) persisted in 0.01% CtE + DF, confirming accelerated acidification probably due to fruit sugars.

Titratable acidity (TA_0_) on day 0 ranged from 0.541 to 0.828 g lactic acid (LA)/100 g, with the lowest TA in the phytosome sample (0.541 ± 0.002 g LA/100 g) and the highest in 0.15% CtE + DF (0.828 ± 0.001 g LA/100 g, *p* < 0.05). After 7 days (TA_7_), the control yogurt exhibited the highest acidity increase (0.850 ± 0.029 g LA/100 g, *p* < 0.05). CtE and DF-fortified samples ranged from 0.757 to 0.801 g LA/100 g, while the phytosome sample remained the lowest (0.679 ± 0.001 g LA/100 g, *p* < 0.05), confirming phytosome encapsulation mitigates acidification over time [37]. These values align with those observed in *Caulerpa racemosa*-fortified yogurts (0.64–0.85%) [41].

The decrease in pH and the increase in acidity over time resulted from the metabolism of lactic acid bacteria, including lactose hydrolysis and organic acid production [44]. Acidity values (0.541–0.850% lactic acid) remained within the Codex Alimentarius standard range (0.60–1.5%) [45].

Ghorbanzade et al. [37] evaluated pH and acidity changes in yogurt samples prepared with nano-encapsulated fish oil over 21 days at 4 °C and similarly observed that control yogurts exhibited the sharpest pH decline, while fortified samples showed slower acidification, consistent with our findings. Their study also confirmed that phytosome encapsulation helped regulate pH reduction and acidity increase, likely by moderating bacterial metabolism and acid production. Additionally, they reported that higher fat content contributed to increased acidity, possibly due to interactions with fermentation dynamics [37].

Lipid oxidation was assessed to evaluate the oxidative stability of the different yogurt formulations. Oxidation in dairy products can lead to off-flavors and reduced shelf life, making it a fundamental parameter for assessing yogurt quality [46].

Lipid oxidation was undetectable (n.d.) in the control yogurt on day 7, while fortified samples exhibited significant peroxide value (PV) increases (*p* < 0.05). The highest PV (0.0483 ± 0.0044 meq O_2_/100 g) was found in 0.15% CtE + DF, indicating higher oxidative susceptibility at elevated extract concentrations. The lowest PV (0.00635 ± 0.00032 meq O_2_/100 g, *p* < 0.05) in phytosome-fortified yogurt suggests that encapsulation significantly reduces lipid oxidation. Lipid peroxidation inhibition probably results from bioactive antioxidant activity (e.g., polyphenols in seaweed extracts) and membrane-stabilizing effects, which restrict oxygen penetration into lipid bilayers, slowing oxidation [47]. However, higher CtE concentrations increased oxidative susceptibility, likely due to the lipid content of algae (2.2 ± 0.1 g/100 g) [14], while nanoencapsulation effectively protected unsaturated fatty acids despite raising total lipid content [48].

Our findings align with previous studies on nanoencapsulation’s role in preserving lipid stability. Ghorbanzade et al. [37] observed that peroxide values increased significantly in yogurts with unencapsulated fish oil. At the same time, nanoencapsulated samples maintained stable PV (~0.6 meq/kg) over 21 days, confirming encapsulation’s protective barrier against oxidation.

These results confirm that phytosome encapsulation enhances oxidative stability, preserving unsaturated fatty acids and extending the shelf life of fortified dairy products.

#### 2.3.5. Syneresis, Moisture, Total Solids, Organic, and Ash Contents in Yogurts

The physicochemical properties of the yogurt samples, including syneresis, moisture content, total solids, organic content, and ash percentage, are summarized in Table 3. Significant differences (*p* < 0.05) were observed among formulations, particularly in syneresis and total solids, which were influenced by the addition of CtE, dehydrated fruit (DF), and phytosome encapsulation.

Syneresis, a key indicator of whey separation and structural stability, varied significantly among samples (*p* < 0.05). The lowest values were observed in the control (19.8%), 0.01% CtE + DF (18.2%), and 0.01% Phytosome (17.2%), indicating better water retention and gel stability. In contrast, the highest syneresis occurred in 0.15% CtE (52.5%) and 0.15% CtE + DF (39.6%), suggesting that higher CtE concentrations disrupt the protein network, reducing water-holding capacity (WHC) and increasing whey separation [36].

These findings align with studies reporting that plant extracts can stabilize or weaken casein gels depending on concentration and protein interactions [49]. Additionally, phytosome encapsulation and increased total solids (via DF) enhanced WHC and reduced syneresis, likely due to emulsifier-stabilized water absorption and a reinforced protein matrix [36].

Moisture content significantly differed among formulations (87.9–90.4%, *p* < 0.05), with the lowest levels in dehydrated fruit-containing samples, likely due to the fruit’s ability to absorb moisture from the yogurt, reducing overall water retention [50].

Total solids, which reflect the sum of organic and inorganic components, influence yogurt’s nutritional density and texture. The lowest values were found in control (9.92%) and 0.01% CtE (9.63%). In comparison, the highest (11.8–12.1%, *p* < 0.05) were observed in CtE + DF and phytosome-containing samples, likely due to dehydrated fruit’s fiber and sugar content, which also contributes to lower moisture content. These findings align with studies showing that fruit fortification increases dry matter, improving texture and reducing syneresis [51,52].

Organic content was the highest in control (94.8%) and slightly but significantly lower (92.2–94.1%) in fortified samples (*p* < 0.05), likely due to added mineral components from CtE and phytosomes.

Ash content, representing inorganic minerals, ranged from 5.20% to 7.82%, with the highest values in phytosome-containing samples. This reflects the mineral richness of seaweed extracts, especially in calcium, potassium, and magnesium [53].

#### 2.3.6. Mineral Composition (Ca, K, Mg and Na)

The mineral composition of the yogurt samples, including calcium (Ca), potassium (K), magnesium (Mg), sodium (Na), and salt (NaCl) content, is presented in Table 4. Significant differences (*p* < 0.05) were observed for potassium, sodium, and salt content, while calcium and magnesium remained relatively stable across formulations.

Calcium levels ranged from 96.2 to 113 mg/100 g, with the 0.01% phytosome sample exhibiting the highest concentration (113 ± 10 mg/100 g, *p* < 0.05). This suggests a potential contribution of phytosome encapsulation to calcium retention. Magnesium content remained relatively unchanged across formulations (19.3–22.9 mg/100 g, *p* > 0.05), indicating that CtE, DF, and phytosomes did not significantly impact Mg presence in yogurt.

Significant increases in potassium (*p* < 0.05) were observed in DF-containing formulations, with 0.01% CtE + DF (144 ± 3 mg/100 g) and 0.15% CtE + DF (137 ± 2 mg/100 g) showing the highest levels. These values were significantly higher than those in the control (104 ± 19 mg/100 g) and 0.01% CtE (93.4 ± 4.3 mg/100 g), suggesting that dehydrated fruit contributed to potassium enrichment, consistent with previous findings in fruit-fortified dairy products [54].

Sodium levels remained similar across formulations (75.7–87.5 mg/100 g, *p* > 0.05), indicating that CtE and phytosome encapsulation did not significantly alter Na content. However, salt content was significantly lower in DF-fortified samples (189–192 mg/100 g) compared to the control (219 mg/100 g), likely due to interactions between fruit-derived bioactives and ionic components affecting mineral solubility [55].

The mineral composition of these yogurts aligns with reported values for natural yogurts: Ca (129–155 mg/100 g), K (98.0–165 mg/100 g), Mg (10.6–15.0 mg/100 g), and Na (81.0–141 mg/100 g) [56]. While seaweed is generally rich in sodium [57], CtE in these formulations did not significantly increase the Na content.

The produced yogurts contribute modestly to the recommended daily intakes (RDAs) of sodium (3.78–4.38%), potassium (2.67–4.12%), and magnesium (6.43–7.62%). In contrast, calcium levels contribute more significantly (12.8–15.0%) than commercial natural yogurts (120 mg/100 g). The Na/K ratio remained below 1.0 in all samples (0.53–0.91), an important dietary metric associated with reduced cardiovascular disease risk [58].

These findings highlight the potential of DF and phytosome encapsulation to influence mineral retention and enhance the nutritional value of yogurt formulations.

#### 2.3.7. Total Lipids, Proteins, Total Sugars, and Energetic Value

The nutritional composition of the yogurt formulations is summarized in Table 5, showing significant differences (*p* < 0.05) across all measured parameters, particularly total lipid content, which was notably influenced by CtE, dehydrated fruit (DF), and phytosome encapsulation.

Total lipids ranged from 0.794% to 5.35%, with the lowest values in 0.01% CtE yogurt (0.79%) and the highest in phytosome-encapsulated samples (5.35%, *p* < 0.05). This increase is attributed to the phospholipid-based encapsulation matrix, consistent with studies reporting elevated fat content in nanoencapsulated bioactives with enhanced oxidative stability [37]. Lower CtE concentrations slightly reduced fat content, possibly due to fat-binding interactions with bioactive compounds [59]. In contrast, higher CtE levels (0.15%) and DF addition increased lipid content (1.89%), likely due to fruit-derived lipids [60].

Protein content ranged from 3.17% in the control to 4.45% in phytosome-encapsulated yogurt, with significantly higher values observed in all fortified formulations (*p* < 0.05). Notably, 0.15% CtE + DF and phytosome samples reached 4.40% and 4.45%, respectively. This increase is likely due to interactions between seaweed bioactives and casein micelles, enhancing protein retention and structural stability [60,61]. Yogurts with 0.01% CtE and 0.01% CtE + DF showed moderate increases (3.54% and 3.75%, respectively), while 0.15% CtE reached 4.28%. The high protein values also reflect green algae’s naturally high protein content (10–47% dry weight) [12] and the protective effect of nanoencapsulation during fermentation [36]. As all fortified samples exceeded typical commercial yogurt protein levels (~3.20 g/100 g), these formulations show strong potential as high-protein functional yogurts [62].

Total sugar content ranged from 1.27% to 3.00%, with significantly lower values in 0.01% CtE + DF (1.27%) and 0.15% CtE (1.37%) compared to the control (2.10%, *p* < 0.05). This reduction likely reflects increased carbohydrate metabolism by lactic acid bacteria in the presence of CtE and fruit polyphenols [63]. In contrast, the phytosome-encapsulated sample showed the highest sugar content (3.00%), possibly due to the encapsulation matrix contributing additional carbohydrates or altering fermentation kinetics [36,42,43]. These findings suggest that CtE and DF can reduce sugar content, supporting the development of low-sugar formulations, while phytosome-containing yogurts may require optimization to manage sugar levels. Compared to commercial yogurts (~4.6 g/100 g), the sugar content in all samples remained lower.

Caloric content varied significantly (27.3–78.0 kcal/100 g), with the lowest values in 0.01% CtE (27.3 kcal) and the highest in phytosome formulations (78.0 kcal, *p* < 0.05). The increased energy value in phytosome-encapsulated yogurts aligns with higher lipid content from the encapsulation system. Compared to commercial yogurts (~3% of daily energy intake), the phytosome formulation contributed 3.90% of the reference daily intake (RDI), whereas other formulations ranged from 1.37% to 2.16%.

Incorporating phytosomes significantly increased yogurt’s caloric content, mainly due to the lipid-based composition of the encapsulation matrix. While low-calorie products are gaining popularity, consumer preferences are also shifting towards functional foods that provide improved bioavailability of bioactive compounds and health benefits. Studies indicate that consumers are willing to accept a moderate increase in caloric content if the product offers added functional properties, such as improved nutrient absorption, oxidative stability, and gut health benefits [64,65].

Furthermore, product formulation strategies can balance this caloric increase by adjusting portions, modifying sugar and fat content, or targeting health-conscious consumers seeking premium functional dairy products. Similar trends have been observed in fortified yogurts and nanoencapsulated bioactives where enhanced nutritional benefits offset higher caloric values [37,66].

These findings confirm that phytosome encapsulation significantly increases lipid and caloric content, while CtE and DF fortification enhance protein levels and reduce sugars. Together, these formulations show promise for functional dairy applications and can be positioned as high-value products for consumers seeking health benefits and innovative yogurt options.

## 3. Material and Methods

### 3.1. Reagents

L-α-phosphatidylcholine (egg yolk, Type XI-E, 100 mg/mL in chloroform, ≥99%, solution), human ApoE3 (recombinant, expressed in *E. coli*, ≥90% (SDS-PAGE), (HPLC), acetylthiocholine iodide ≥ 98% (TLC), butyrylthiocholine iodide ≥ 98%, butyrylcholinesterase from equine serum, acetylcholinesterase from electric eel, 5,5′-dithiobis(2-nitrobenzoic acid), Trizma^®^ (St. Louis, MO, USA), ≥99.9% (titration), crystalline, bovine serum albumin, cold ethanol fraction, pH 5.2, ≥96%, 1-octanol ACS reagent, suitable for UV/vis spectroscopy, ≥99.5% (GC), 6-hydroxy-2,5,7,8-tetramethylchroman-2-carboxylic acid (Trolox), disodium fluorescein, potassium phosphate monobasic (KH_2_PO_4_), potassium phosphate dibasic trihydrate (K_2_HPO_4_·3H_2_O), and 2′,2′-azobis (2-amidinopropane) dihydrochloride (AAPH) were acquired from Sigma Chemicals Co. (). Anhydrous absolute ethanol and 96% sulfuric acid, RPE (for analysis—ISO) were acquired from Carlo Erba Reagents (Chau. du Vexin, Val-de-Reuil, France); ACS BASIC CL0217 chloroform, stabilised with ethanol from Scharlab (Barcelona, Spain), KBr—potassium bromide (IR) PAI and tablets for Kjeldahl (Catalyst with 0.3% CuSO_4_·5H_2_O) from Panreac (Darmstadt, Germany); DSPE-PEG(2000) amine (chloroform 1,2-distearoyl-sn-glycero-3-phosphoethanolamine-N-[amino (polyethylene glycol)—2000]), from Avanti Polar Lipids (Alabaster, AL, USA); soluble starch GR ISO. CAS 9005-84-9, pH 6.0–7.5, sodium thiosulfate pentahydrate 99.5% for analysis, both from Merck (Darmstadt, Germany); potassium iodide RPE-ACS, sodium hydroxide AGR, and low metal micro-pearls, from Labbox (Barcelona, Spain); potassium hydrogenophthalate, ≥99.5%, from Honeywell (Charlotte, NC, USA). Potassium persulfate, and 2,2-azinobis (3-ethylbenzothiazoline-6-sulfonic acid) di-ammonium salt (ABTS) were purchased from Merck (Darmstadt, Germany).

### 3.2. Samples and Extraction

*C. tomentosum* extract was produced by subcritical water extraction as described by Soares, C. et al. [14]. In this study, only the E4 fraction was used, herein named CtE.

### 3.3. Box–Behnken Factorial Design

A Box–Behnken design with three independent variables [A—time (1–4 h), B—temperature (25–60 °C), and C—ratio CtE:phosphatidylcholine (1:1–1:4)] was applied to obtain the best conditions to maximize the phytosome complex formation. A total of 15 runs were performed, considering 3 central points. The software Design Expert (version 11, Stat-Ease Inc., Minneapolis, MN, USA) was used for experimental design, data analysis, and model building. After determining the model equation, Derringer’s desirability function was used to maximize the modeled responses.

### 3.4. Phytosome Production and Characterization

The phytosomes were produced using the optimal conditions of temperature, time, and ratio CtE:phosphatidylcholine. Phytosome production started by mixing 3 mg of CtE and 30 µL of L-α-phosphatidylcholine (100 mg/mL in chloroform). After drying the chloroform under nitrogen flow, 3 mL of ethanol was added. To allow the reaction to occur, the mixture was placed in a water bath at 59 °C for 1 h. After this period, ethanol was evaporated under nitrogen, and 3 mL of chloroform was added to the dried complex. Then, the mixture was filtered twice through PTFE membranes (0.22 µm) to remove non-complexed extract. Phytosomes were stored dried, after chloroform evaporation, and used in a few days. The complex was further functionalized with 3% ApoE and 2.5% DSPE-PEG(2000) amine. Afterward, they were characterized by dynamic light scattering (DLS), UV spectrophotometry, octanol/water partition coefficient (Kow), differential scanning calorimetry (DSC), and Fourier transform infrared spectroscopy (FTIR), according to established procedures [30,33].

DLS analysis was performed in a Malvern Zetasizer ZS instrument (Worcestershire, UK), using disposable polystyrene cuvettes for each sample. Prior to each analysis, the samples were homogenized using an ultrasound bath. The intensity-distribution size was obtained with the Malvern Zetasizer v7.11 software.

CtE, L-α-phosphatidylcholine and phytosomes UV-vis spectra were traced in a UV-vis Shimadzu spectrophotometer (model UV-2101PC, Tokyo, Japan). For that, samples were diluted in chloroform and submitted to ultrasound to ensure correct solubilization. The UV-vis spectra were traced from 200 to 700 nm.

The solubility in water or in organic solvents was determined by adding 10 mL of water and 10 mL of n-octanol to the complex or to CtE, in a decantation funnel. The mixture was agitated for 24 h at room temperature. After phase separation, both phases were centrifuged for 20 min at 1000 rpm. The supernatant was filtered through PTFE membranes (0.22 µm). Sample concentration was determined at 280 nm (characteristic wavelength of CtE) in a microplate reader (BioTek Synergy HTX Multimode Reader, Winooski, VT, USA, EUA) using the calibration curves traced for CtE dissolved in water and CtE dissolved in n-octanol. The K_ow_ partition coefficient was calculated according to Equation (2):

K_ow_ = P = C_o_/C_w_ (2)

where C_o_ = concentration in the n-octanol phase and C_w_ = concentration in the aqueous phase.

To obtain L-phosphatidylcholine, CtE, and phytosomes thermograms, 3.0 ± 0.2 mg of each sample was placed into alumina crucibles. An empty alumina crucible was used as a reference. Thermograms and DSC results were obtained using a thermal analyzer STA 449 F3 Jupiter^®^ (NETZSCH, Selb, Germany) with NETZSCH Proteus^®^ 5.2 software. The temperature program applied ranged from 25 °C to 1000 °C, with a rate of 20 °C per minute under a nitrogen flow of 50 mL/min.

For FTIR analysis, CtE was grounded with KBr (1:100) in a mortar. Transparent ultrapure pellets were formed by applying a 10 Ton/nm^2^ force. For L-α-phosphatidylcholine and phytosomes, producing KBr-sample pellets was not feasible, so the samples were directly placed between NaCl salt plates. The samples were analyzed using a Nicolet 6700 FT-IR (Thermo Fisher Scientific, Waltham, MA, USA) with OMNIC^TM^ Spectra 8.3 software. The spectrum was obtained with a resolution of 4 cm^−1^ and analyzed within the wavenumber range of 400–4000 cm^−1^.

### 3.5. Functional Yogurt Preparation and Characterization

The yogurt formulation followed a predefined recipe, was fermented for approximately 12 h in a yogurt maker, and then stored at 4 °C. A commercial ultra-pasteurized semi-skimmed milk containing 16 g of lipids, 49 g of sugars, 34 g of proteins, 1 g of salt, and 1.2 g of calcium per liter was used. Additionally, a commercial natural yogurt was employed as a source of lactic acid bacteria for fermentation, with 2.5 g of lipids, 4.6 g of sugars, 3.2 g of proteins, 0.14 g of salt, and 120 mg of calcium per 100 g. A total of ten yogurt samples were prepared, containing varying concentrations of the CtE: 0%, 0.005%, 0.01%, 0.05%, 0.1%, and 0.2%. Additional formulations included phytosome-encapsulated CtE at 0.01% and 0.15% and yogurts with 0.01% and 0.15% CtE combined with powdered dehydrated blackberry and raspberry. Physicochemical properties, such as pH, acidity, syneresis [37], moisture, total solids, organic and inorganic content [67], peroxides [68], proteins [69], total lipids [70], sugar content [71], and mineral composition [72], were analyzed according to established procedures. The in vitro inhibition capacity of the brain enzyme AChE and BuChE activities for each yogurt was measured according to a modified Ellman assay [14,73]. For antioxidant activity, the 2,2′-azino-bis(3-ethylbenzothiazoline-6-sulfonic acid radical (ABTS^•+^) [74] and the oxygen radical absorbance capacity (ORAC) [14] assays were used. Trolox was used as standard in both assays. The results were expressed as mg Trolox equivalents (TE) per 100 g yogurt (mg TE/100 g) for ABTS and μmol Trolox equivalents per 100 g yogurt (μmol TE/100 g). For all the extracts, triplicate measurements were made.

For the sensorial analysis, a restricted group of informed, untrained, informal tasters was selected to verify possible alterations in fortified yogurts’ aroma, taste, color, and texture using the affective method expressed by each taster’s opinion.

### 3.6. Statistical Analysis

The data are presented as mean ± standard deviation and were evaluated using one-way ANOVA. Tukey’s HSD post hoc test (*p* < 0.05) was employed to assess group differences. Analyses were conducted using SPSS (Version 29.0.2, IBM Corp., Armonk, NY, USA, 2023).

## 4. Conclusions

Phytosomes complexed with a neuroprotective seaweed fraction were successfully developed at the optimal conditions of temperature 59 °C, time 1 h, and ratio CtE:phosphatidylcholine of 1:1, offering a promising functional food ingredient designed for the elderly. FTIR and DSC analyses confirmed the formation of a phyto-phospholipid complex, while DLS analysis demonstrated a small particle size and low polydispersity, indicating favorable dispersion properties. Additionally, the octanol/water partition coefficient was higher for the phytosome complex than for the isolated CtE, suggesting improved bioavailability. Given the increasing need for neuroprotective dietary interventions in aging populations, these phytosomes hold the potential for being incorporated into functional yogurts and other food matrices aimed at supporting cognitive health in the elderly. Future research will focus on evaluating their stability, bioaccessibility, and long-term effects on cognitive function in aging individuals. Further studies, including in vitro digestion models and release profile assessments, are necessary to evaluate the liberation of the CtE under physiological conditions and optimize the formulation for enhanced functional benefits.

## Figures and Tables

**Table 1:** Antioxidant activity (ABTS and ORAC) of yogurt formulations containing CtE and phytosomes.

Samples	ABTS (mg TE/100 g)	ORAC (μmol TE/100 g)
Control (0%)	13.5 ± 0.3 ^a^	62.1 ± 0.3 ^a^
0.01% CtE	11.1 ± 0.1 ^a^	118 ± 1 ^b^
0.01% CtE + DF	24.2 ± 1.0 ^c^	245 ± 1 ^e^
0.15% CtE	18.4 ± 1.6 ^b^	162 ± 3 ^c^
0.15% CtE + DF	27.0 ± 0.7 ^d^	167 ± 1 ^d^
0.01% Phytosomes	12.9 ± 1.2 ^a^	60.0 ± 0.1 ^a^

Within columns, different letters correspond to significantly different values (*p* < 0.05).

**Table 2:** Effect of seaweed extracts, dehydrated fruits, and phytosome encapsulation on pH, titratable acidity, and peroxide value in yogurt over 7 days at 4 °C.

Samples	pH_0_	pH_7_	TA_0_ (g LA/100 g Yogurt)	TA_7_ (g LA/100 g Yogurt)	PV_7_ (meqO_2_/100 g Yogurt)
Control (0%)	4.36 ± 0.01 ^B,d^	4.31 ± 0.01 ^A,d^	0.708 ± 0.003 ^A,b^	0.850 ± 0.029 ^B,b^	n.d.
0.01% CtE	4.37 ± 0.01 ^A,d^	4.33 ± 0.01 ^A,e^	0.683 ± 0.028 ^A,b^	0.773 ± 0.089 ^A,a,b^	0.0267 ± 0.0012 ^b^
0.01% CtE + DF	4.26 ± 0.01 ^B,a^	4.23 ± 0.01 ^A,a^	0.820 ± 0.004 ^B,c^	0.757 ± 0.022 ^A,a,b^	0.0254 ± 0.0015 ^b^
0.15% CtE	4.31 ± 0.01 ^B,c^	4.29 ± 0.01 ^A,c^	0.816 ± 0.001 ^B,c^	0.801 ± 0.001 ^A,b^	0.0418 ± 0.0033 ^c^
0.15% CtE + DF	4.28 ± 0.01 ^A,b^	4.24 ± 0.01 ^B,b^	0.828 ± 0.001 ^B,c^	0.800 ± 0.001 ^A,b^	0.0483 ± 0.0044 ^c^
0.01% Phytosomes	4.40 ± 0.01 ^B,e^	4.37 ± 0.01 ^A,f^	0.541 ± 0.002 ^A,a^	0.679 ± 0.001 ^B,a^	0.00635 ± 0.00032 ^a^

0: initial time; 7: day 7; TA: titratable acidity; LA: lactic acid equivalents. Values are presented as mean ± standard deviation. Different lowercase letters in the same column indicate significant differences between samples (*p* < 0.05). Different uppercase letters in the same row indicate significant differences over time for pH and titratable acidity (*p* < 0.05).

**Table 3:** Physicochemical properties and proximate composition of yogurt samples with different formulations.

Samples	Syneresis (%)	Moisture (%)	Total Solids (%)	Organic Content (%)	Ash (%)
Control (0%)	19.8 ± 1.4 ^a^	90.1 ± 0.1 ^c^	9.92 ± 0.08 ^a^	94.8 ± 0.2 ^c^	5.20 ± 0.25 ^a^
0.01% CtE	30.1 ± 2.4 ^b^	90.4 ± 0.3 ^c^	9.63 ± 0.30 ^a^	94.1 ± 0.2 ^b^	5.87 ± 0.24 ^b^
0.01% CtE + DF	18.2 ± 0.7 ^a^	87.9 ± 0.1 ^a^	12.1 ± 0.1 ^c^	92.6 ± 0.4 ^a^	7.43 ± 0.38 ^c^
0.15% CtE	52.5 ± 3.2 ^d^	88.9 ± 0.1 ^b^	11.1 ± 0.1 ^b^	92.6 ± 0.2 ^a^	7.38 ± 0.15 ^c^
0.15% CtE + DF	39.6 ± 3.6 ^c^	87.9 ± 0.1 ^a^	12.1 ± 0.1 ^c^	92.3 ± 0.3 ^a^	7.73 ± 0.27 ^c^
0.01% Phytosomes	17.2 ± 1.0 ^a^	88.2 ± 0.1 ^a^	11.8 ± 0.1 ^c^	92.2 ± 0.1 ^a^	7.82 ± 0.01 ^c^

Values are presented as mean ± standard deviation. Different lowercase letters in the same column indicate significant differences between samples (*p* < 0.05).

**Table 4:** Mineral Composition of Yogurt Samples with Different Formulations.

Samples	Ca (mg/100 g Yogurt)	K (mg/100 g Yogurt)	Mg (mg/100 g Yogurt)	Na (mg/100 g Yogurt)	Salt (mg/100 g Yogurt)
Control (0%)	96.8 ± 3.9 ^a^	104 ± 19 ^a,b^	21.6 ± 1.1 ^a^	87.5 ± 16.9 ^a^	219
0.01% CtE	96.2 ± 3.0 ^a^	93.4 ± 4.3 ^a^	22.6 ± 2.3 ^a^	85.3 ± 1.9 ^a^	213
0.01% CtE + DF	96.4 ± 3.7 ^a^	144 ± 3 ^c^	22.9 ± 0.2 ^a^	76.7 ± 1.8 ^a^	192
0.15% CtE	106 ± 1 ^a,b^	121 ± 4 ^b,c^	22.1 ± 0.3 ^a^	84.7 ± 4.5 ^a^	212
0.15% CtE + DF	103 ± 4 ^a,b^	137 ± 2 ^c^	22.7 ± 0.4 ^a^	75.7 ± 0.8 ^a^	189
0.01% Phytosomes	113 ± 10 ^b^	99.4 ± 7.4 ^a,b^	19.3 ± 2.0 ^a^	75.9 ± 6.4 ^a^	190

Values are presented as mean ± standard deviation. Different lowercase letters in the same column indicate significant differences between samples (*p* < 0.05). Recommended daily intakes of minerals: Ca: 750 mg/day; K: 3500 mg/day; Mg: 300 mg/day; Na; 2000 mg/day (https://www.efsa.europa.eu/en/topics/topic/dietary-reference-values, accessed on 20 January 2023). Recommended maximum daily salt (NaCl) intake: <5000 mg/day, calculated from 2000 mg Na × 2.5 (https://www.who.int/data/gho/indicator-metadata-registry/imr-details/3082, accessed on 3 March 2025).

**Table 5:** Nutritional composition of yogurt samples with different formulations.

Samples	Total Lipids (%)	Total Sugars (%)	Total Protein (%)	Caloric Content (kcal)
Control (0%)	1.05 ± 0.09 ^a,b^	2.10 ± 0.19 ^b^	3.17 ± 0.29 ^a^	30.6
0.01% CtE	0.794 ± 0.056 ^a^	1.51 ± 0.11 ^a^	3.54 ± 0.25 ^a,b^	27.3
0.01% CtE + DF	0.921 ± 0.037 ^a,b^	1.27 ± 0.05 ^a^	3.75 ± 0.15 ^a,b,c^	28.4
0.15% CtE	1.49 ± 0.10 ^b,c^	1.37 ± 0.10 ^a^	4.28 ± 0.30 ^b,c^	36.0
0.15% CtE + DF	1.89 ± 0.09 ^c^	2.14 ± 0.11 ^b^	4.40 ± 0.22 ^c^	43.2
0.01% Phytosomes	5.35 ± 0.48 ^d^	3.00 ± 0.27 ^c^	4.45 ± 0.40 ^c^	78.0

Values are presented as mean ± standard deviation. Different lowercase letters in the same column indicate significant differences between samples (*p* < 0.05). Caloric content was calculated using the Atwater general factor system where the energy values are 4.0 kcal/g for protein, 9.0 kcal/g for fat, and 4.0 kcal/g for sugars (https://www.fao.org/4/y5022e/y5022e04.htm#fn9, accessed on 21 January 2025). A daily caloric intake of 2000 kcal was adopted as a reference value, in line with general dietary guidelines provided by EFSA (https://www.efsa.europa.eu/en/topics/topic/dietary-reference-values, accessed on 4 March 2025).

## Data Availability

The datasets analyzed in the current study are available from the corresponding author upon reasonable request.

## Supplementary Materials

The following supporting information can be downloaded at: https://www.mdpi.com/article/10.3390/md23040147/s1, Table S1: Entrapment efficiency (%) as a function of the three independent variables (t = time (h); T = temperature (°C); Ratio (CtE:PC) = ratio (CtE:L-α-phosphatidylcholine)); Table S2: ANOVA analysis and statistical parameters of the reduced cubic model for the entrapment efficiency (%) using three parameters (A, B and C); Figure S1: Diagnostic graphs. A—Predicted vs Actual values; B—Normal Plot of Residuals; C—Residuals vs Predicted; D—Residuals vs Run; E—Residuals vs Time; F—Residuals vs Temperature; G—Residuals vs CtE:PC ratio.
