# Supplementary material for: Optimization of Nanoencapsulation of Codium tomentosum Extract and Its Potential Application in Yogurt Fortification
Source: Mar Drugs. 2025 Mar 28;23(4):147. doi: 10.3390/md23040147 (PMC12028962; doi:10.3390/md23040147)
Supplement: Supplementary file 1 [file marinedrugs-23-00147-s001.zip › marinedrugs-3532353-supplementary.pdf]

Supplementary material

# Optimization of Nanoencapsulation of *Codium tomentosum* Extract and Its Potential Application in Yogurt Fortification

**Micaela Costa [1], Cristina Soares [1,*], Aurora Silva [1,2], Maria Fátima Barroso [1], Pedro Simões [3], Mariana Ferreira [4], Paula Gameiro [4], Clara Grosso [1,*] and Cristina Delerue-Matos [1]**

[1] REQUIMTE/LAQV, Instituto Superior de Engenharia do Porto, Instituto Politécnico do Porto, Rua Dr. António Bernardino de Almeida 431, 4249-015 Porto, Portugal; 1200125@isep.ipp.pt (M.C.); mass@isep.ipp.pt (A.S.); mfb@isep.ipp.pt (M.F.B.); cmm@isep.ipp.pt (C.D.-M.)
[2] Department of Analytical Chemistry and Food Science, Nutrition and Food Group (NuFoG), Instituto de Agroecoloxía e Alimentación (IAA)—CITEXVI, Universidade de Vigo, 36310 Vigo, Spain
[3] LAQV, REQUIMTE, Departamento de Química, Faculdade de Ciências e Tecnologia, Universidade NOVA de Lisboa, Quinta da Torre, 2829-516 Caparica, Portugal; pcs@fct.unl.pt
[4] LAQV, REQUIMTE, Departamento de Química e Bioquímica, Faculdade de Ciências da Universidade do Porto, Rua do Campo Alegre, 4169-007 Porto, Portugal; mariana.ferreira@fc.up.pt (M.F.); agsantos@fc.up.pt (P.G.)
* Correspondence: cds@isep.ipp.pt (C.S.); claragrosso@graq.isep.ipp.pt (C.G.)

**Table S1.** Entrapment efficiency (%) as a function of the three independent variables (t = time (h); T = temperature (°C); Ratio (CtE :PC) = ratio (CtE : L-$\alpha$-phosphatidylcholine)).

**Table S2.** ANOVA analysis and statistical parameters of the reduced cubic model for the entrapment efficiency (%) using three parameters (A, B and C).

**Figure S1.** Diagnostic graphs. A - Predicted vs Actual values; B – Normal Plot of Residuals; C - Residuals vs Predicted; D - Residuals vs Run; E - Residuals vs Time; F - Residuals vs Temperature; G - Residuals vs CtE :PC ratio.

**Table S1.** Entrapment efficiency (%) as a function of the three independent variables (t = time (h); T = temperature (°C); Ratio (CtE :PC) = ratio (CtE : L-$\alpha$-phosphatidylcholine)).

| Run | t (h) | T (°C) | Ratio (CtE:PC) | Actual entrapment efficiency (%) | Predicted Entrapment efficiency (%) |
|---|---|---|---|---|---|
| 1 | 1 | 25 | 1:2.5 | 26.52 | 27.80 |
| 2 | 4 | 25 | 1:2.5 | 39.17 | 38.28 |
| 3 | 1 | 60 | 1:2.5 | 45.70 | 46.98 |
| 4 | 4 | 60 | 1:2.5 | 44.81 | 43.92 |
| 5 | 1 | 42.5 | 1:1 | 43.05 | 41.77 |
| 6 | 4 | 42.5 | 1:1 | 37.42 | 38.31 |
| 7 | 1 | 42.5 | 1:4 | 25.19 | 23.91 |
| 8 | 4 | 42.5 | 1:4 | 33.92 | 34.81 |
| 9 | 2.5 | 25 | 1:1 | 31.94 | 31.74 |
| 10 | 2.5 | 60 | 1:1 | 41.40 | 41.20 |
| 11 | 2.5 | 25 | 1:4 | 45.67 | 45.47 |
| 12 | 2.5 | 60 | 1:4 | 37.58 | 37.38 |
| 13 | 2.5 | 42.5 | 1:2.5 | 36.63 | 34.41 |
| 14 | 2.5 | 42.5 | 1:2.5 | 33.38 | 34.41 |
| 15 | 2.5 | 42.5 | 1:2.5 | 32.43 | 34.41 |

**Table S2.** ANOVA analysis and statistical parameters of the reduced cubic model for the entrapment efficiency (%) using three parameters (A, B and C).

| Source | Sum of squares | df | Mean squares | F-value | p-value |
|---|---|---|---|---|---|
| **Model** | 571.82 | 10 | 57.18 | 11.59 | 0.0153 |
| A-Time | 27.60 | 1 | 27.60 | 5.59 | 0.0772 |
| B-Temperature | 0.4692 | 1 | 0.4692 | 0.0951 | 0.7732 |
| C-Ratio | 24.55 | 1 | 24.55 | 4.98 | 0.0895 |
| AB | 45.83 | 1 | 45.83 | 9.29 | 0.0381 |
| AC | 51.55 | 1 | 51.55 | 10.45 | 0.0319 |
| BC | 77.00 | 1 | 77.00 | 15.61 | 0.0168 |
| $A^2$ | 0.3186 | 1 | 0.3186 | 0.0646 | 0.8119 |
| $B^2$ | 76.74 | 1 | 76.74 | 15.55 | 0.0169 |
| $A^2B$ | 68.74 | 1 | 68.74 | 13.93 | 0.0203 |
| $A^2C$ | 122.23 | 1 | 122.23 | 24.77 | 0.0076 |
| **Residual** | 19.74 | 4 | 4.93 | | |
| Lack of fit | 10.03 | 2 | 5.02 | 1.03 | 0.4916 |
| Pure error | 9.70 | 2 | 4.85 | | |
| **Cor total** | 591.56 | 14 | | | |
| **Std. Dev.** | 2.22 | | | **$R^2$** | 0.9666 |
| **Mean** | 36.99 | | | **Adjusted $R^2$** | 0.8832 |
| **C.V. %** | 6.01 | | | **Predicted $R^2$** | 0.4041 |
| | | | | **Adeq Precision** | 12.1256 |

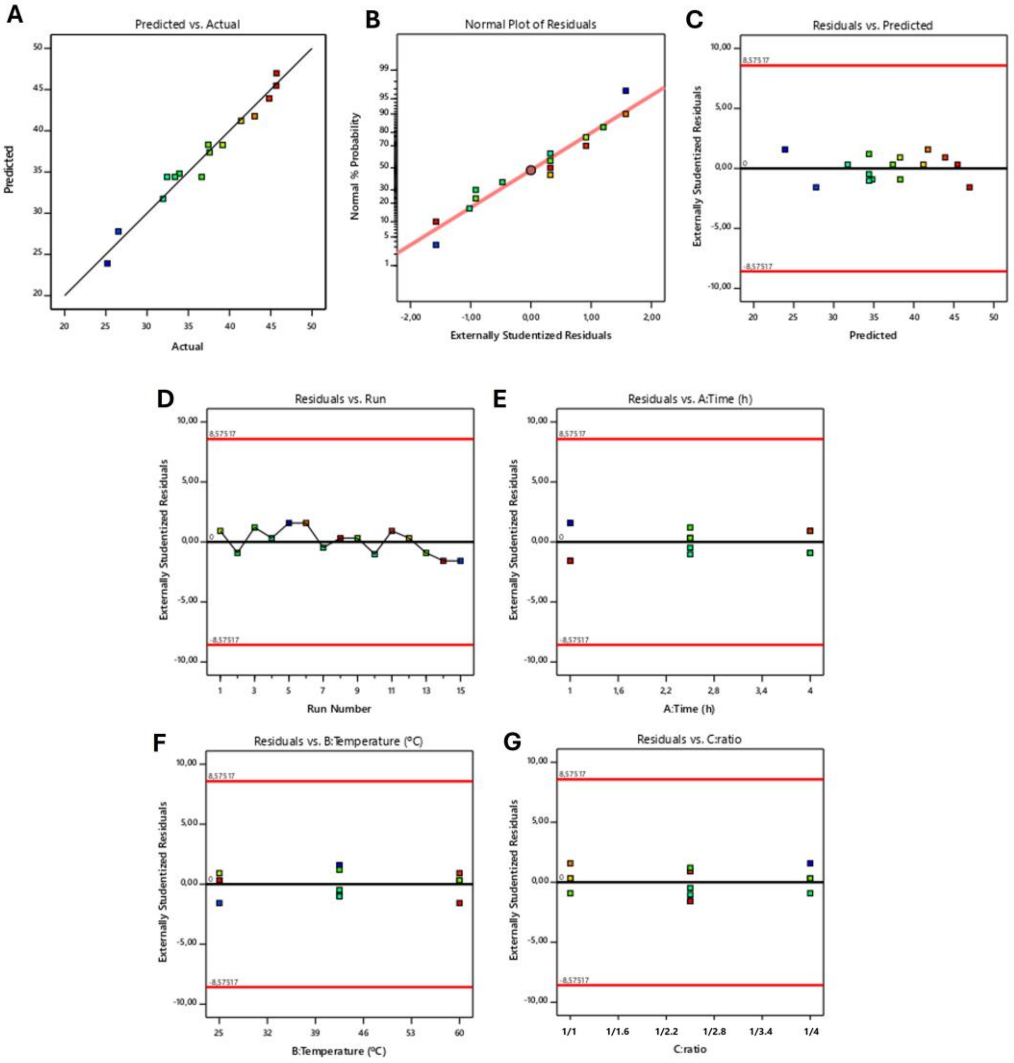


**Figure S1.** Diagnostic graphs. A - Predicted vs Actual values; C - Residuals vs Predicted; D - Residuals vs Run; E - Residuals vs Time; F - Residuals vs Temperature; G - Residuals vs CtE :PC ratio.
